# Gene Therapy in Cardiac Arrhythmias

**Published:** 2006-04-01

**Authors:** Praveen S V, Johnson Francis, Venugopal K

**Affiliations:** Department of Cardiology, Medical College, Calicut, Kerala, India

**Keywords:** Gene therapy, biological pacemakers, AV node modification, long QT syndromes, sarcoplasmic reticulum calcium pump

## Abstract

Gene therapy has progressed from a dream to a bedside reality in quite a few human diseases. From its first application in adenosine deaminase deficiency, through the years, its application has evolved to vascular angiogenesis and cardiac arrhythmias. Gene based biological pacemakers using viral vectors or mesenchymal cells tested in animal models hold much promise. Induction of pacemaker activity within the left bundle branch can provide stable heart rates. Genetic modification of the AV node mimicking beta blockade can be therapeutic in the management of atrial fibrillation. G protein overexpression to modify the AV node also is experimental. Modification and expression of potassium channel genes altering the delayed rectifier potassium currents may permit better management of congenital long QT syndromes. Arrhythmias in a failing heart are due to abnormal calcium cycling. Potential targets for genetic modulation include the sarcoplasmic reticulum calcium pump, calsequestrin and sodium calcium exchanger. Lastly the ethical concerns need to be addressed.

## Introduction

Gene therapy is defined as the transfer of nucleic acids to somatic cells as therapeutically useful molecules. Genetic defects can be corrected or gene products be expressed by gene therapy. This approach has many potential applications, the most obvious being the treatment of inherited monogenic disorders like cystic fibrosis. Human genome has approximately 30,000 genes. The genetic diversity is amplified by alternate splicing of mRNA and post translational modification of proteins. The possible gene targets for arrhythmias is very large. Anti arrhythmic agents act by blocking the ion channels. These antiarrhythmics have poor channel specificity and may cross react with other ion channels. In addition they have poor affinity for the channels and have a narrow therapeutic window. The molecular targets of arrhythmia management are the ion channels and the modulators of ion channels like G proteins [1].

## Vectors for gene therapy

A vector is the vehicle commonly used to introduce the gene to the target cell. Vectors may be RNA or DNA viruses or non viral in nature. Viruses which have the capacity to incorporate themselves in the host genome are used as vectors for gene therapy. The commonly used viral vectors are genetically modified retroviruses, adenoviruses, adeno associated viruses and lentiviruses. These viral vectors are made replication deficient to ensure safety, but requires large amounts of vector particles for efficacy. Non viral vectors based on plasmids, DNA- lipid complexes and naked DNA are also used since they lack foreign proteins and avoid immunological problems. None of the currently available vectors satisfy the criteria of an ideal gene therapeutic system. The feasibility of gene transfer has been demonstrated in both animals and humans.

In contradistinction to the experimental laboratory, the extent of gene transfer and expression is low in clinical settings. The period during which a newly introduced gene is expressed is variable and differs with the tissue, but is often short. For example, early-generation non-viral vectors express the gene at maximum levels only for a few days [2]. Many adenoviral vectors express the gene for 2-3 weeks [3]. Non viral vectors also have short duration of gene expression. Short duration of gene expression necessitates repeat dosing, although less efficacious. By contrast, expression from adeno-associated viral vectors may not peak for several weeks, but then remain constant in some tissues for several months [4]. Retroviruses produce a long lasting effect by integration of the transfected gene into the host genome [5]

## Methods of gene delivery

The classical methods of vector delivery are direct injection into the myocardium, infusion through the coronary arteries or administration to the epicardium.Various novel methods of transfection have been tried in animal models, including DNA polymer coating on inert materials and subsequent transfer to the atrial myocardium, with sustained gene activity [6]. Intracoronary perfusion is another modality of gene transduction with near complete expression under optimal conditions [7]. The gene transfer efficiency depends on the coronary flow rate, virus concentration, virus exposure time and microvascular permeability. Agents which increase the microvascular permeability have been used to enhance the delivery. Each disease has its own target tissue and the amount of gene product required for treatment. So only few generalizations can be made about the vector selection and the method of gene delivery.

## The need for gene therapy in cardiac arrhythmias

Antiarrhythmic medications suppress arrhythmias, but their systemic effects are often poorly tolerated and their proarrhythmic effects increase mortality. Radiofrequency ablation can cure only a limited number of arrhythmias. Implantable devices can be curative for bradyarrhythmias and lifesaving for tachyarrhythmias, but require a lifetime commitment to repeated procedures, have a significant expense, and may lead to severe complications. The need for new treatment strategies for cardiac arrhythmias has motivated the continuing development of gene therapeutic options Gene therapy may prove to be a less intrusive, long-term solution to arrythmias than pacemakers or antiarrhythmic agents. Gene therapy for arrhythmias is a field still in its infancy.

The ongoing research into gene therapy for cardiac arrhythmias can be briefly discussed under:

1. Biological pacemakers
2. AV nodal modification
3. Long QT syndromes
4. Arrhythmias of cardiac failure
5. Ventricular arrhythmias

## Biological pacemakers

Conventional electronic pacemakers although highly efficacious, have a finite battery life and problems related to lead and circuitry. Biological pacemakers aim a near physiological pacing by molecular or cellular targeting.Biological pacemakers can be cell therapy based or gene therapy based. Strategies have included gene therapy using naked plasmids or viral vectors and cell therapy with both adult human mesenchymal stem cells (hMSCs) and human embryonic stem cells [8,9]. Over the past few years, gene therapy has been used to explore the overexpression of β2-adrenergic receptors, the down-regulation of inward rectifier current, and the overexpression of pacemaker current as potential sources of biological pacemakers [10]. The protein product of HCN2 gene is a candidate for the pacemaker current. Rosen et al from Columbia university have recreated the biological pacemaker by administering the pacemaker gene HCN2 via viral vector or in an hMSC platform to produce pacemaker function in the intact canine heart. Adenovirus containing the HCN2 gene [ad HCN2] on introduction in canine models produced spontaneous rhythms.

Another potential approach to a biological pacemaker is the induction of pacemaker activity within the left bundle branch, thereby providing a ventricular escape rhythm with physiologically acceptable rates. The long-term stability and feasibility of this approach remain to be tested [11]. Adeno viral vectors have been tried in animal models to create pacemaker clones from ventricular myocytes. The inward rectifier current [I_K1_] is responsible for the maintenance of the resting membrane potential. The main subunit of this is Kir 2.1. The Kir 2.1 gene was mutated to make it a dysfunctional channel (a dominant-negative), inserted into an adenoviral vector and delivered to the hearts of guinea pigs. The enhanced automaticity of the ventricular myocytes subsequent to the gene transfer conferred pacemaker like properties [12].

## AV nodal modification

As in case of biological pacemakers, the field is limited to animal studies. The major area of research in gene therapy for tachyarrhythmias now is on AV nodal modification to produce AV nodal block mimicking beta blockade.

Genetic modification of AV node in an intracoronary perfusion model of porcine heart was reported by Donahue et al from John Hopkins University [13]. They infected porcine hearts with Adbetagal (recombinant adenovirus expressing Escherichia coli beta-galactosidase) or with AdGi [adenovirus encoding the Galphai2 subunit]. Galphai2 overexpression suppressed baseline atrioventricular conduction and slowed the heart rate during atrial fibrillation without producing complete heart block. In contrast, expression of the reporter gene beta-galactosidase had no electrophysiological effects. Reporter genes are nucleic acid sequences encoding easily assayed proteins.

AV nodal gene transfer can decrease heart rates in animal models of atrial fibrillation. Inhibitory G protein overexpression can prolong the AV nodal refractory period with slowing of conduction resulting in reduced ventricular rates in atrial fibrillation [14]. In mouse models, cardiac overexpression of adenosine receptor [A(3)AR] resulted in gene dose-dependent AV block and pronounced sinus nodal dysfunction in vivo. These may have future therapeutic implications for SA and AV nodal modification [15]. Focal gene transfer to the AV node to produce a genetic calcium channel blocker has been successful in experimental settings. Over expression of the ras related small G- protein [Gem] in AV node slowed AV nodal conduction controlling the heart rate in atrial fibrillation [16].

## Long QT syndromes ( LQTS)

Sympathetic imbalance was previously thought to be responsible for this disease. Since 1991, 7 LQTS genes have been discovered and more than 300 mutations have been identified to account for the disease. Acquired LQTS, is presumed to be due to the blockade of the rapid component of the delayed rectifier K+ current (I_Kr_). Blockage of the I_Kr_ prolongs the QT interval and increases the dispersion of repolarization, predisposing to torsades de pointes. Molecular genetic analysis could be useful to unravel subclinical mutations or polymorphisms.

Individuals with cardiac potassium channel missense mutation, Q9E-hMiRP1 are predisposed to develop QT prolongation after clarithromycin administration. Experimental studies have demonstrated that cells transfected with plasmid DNA containing Q9E-hMiRP1 have reduced potassium currents on exposure to clarithromycin.

Site specific gene therapy for arrhythmias by transfecting cell clones with the K+ channel genes is a feasible approach to the management of LQTS [17]. Mutated K+ channels resulting in loss of function have been implicated in LQT 1 and 2. The potassium channel alpha subunit genes KCNH2 [HERG] and KCNQ1 [KvLQT1] responsible for I_Kr_ and I_Ks_ respectively are mutated in LQTS. In normal epithelia, KCNE3 [E3] interacts with the KVQT1 [Q1] thereby augmenting the potassium currents. E3 subunit can be genetically expressed in cardiac tissues [where it is normally scarce] to abbreviate the action potential duration and enhance the potassium current.This potentially prevents arrhythmias in LQTS. Adenovirus encoded E3 introduced into guinea pig ventricles shortened QT interval on homogenous transduction, but could be potentially arrhythmogenic if transduction is heterogenous [18].

## Arrhythmias of cardiac failure

Abnormal calcium cycling plays an important role in the genesis of contractile dysfunction and arrhythmias in the setting of heart failure. Genetic modulation of the sarcoplasmic reticulum calcium ATP ase pump [SERCA] can have an ameliorating effect on the arrhythmias of cardiac failure.

Delayed repolarization predisposes the failing heart to ventricular arrhythmias and this represents a logical target for gene therapy. The calcium ATPase SERCA1 was coexpressed with the potassium channel Kir2.1 in guinea pig hearts. Such myocytes had bigger calcium transients and shorter action potentials. In vivo, repolarization was abbreviated, but contractile function remained unimpaired [19]. This shortening of repolarization prevents arrhythmias. Coexpression of SERCA1 prevented the decrease in contractility due to shortening of action potential. This interesting observation is useful in prevention of arrhythmias in the setting of heart failure.

Calsequestrin, the high capacity calcium binding protein expressed in sarcoplasmic reticulum, also positively controls the rate of calcium release during excitation-contraction coupling. Mutations in the calsequestrin gene have been linked to arrhythmias and sudden death. For example, the recessive form of catecholaminergic polymorphic ventricular tachycardia is associated with calsequestrin mutations [20]. Modulation of the gene for calsequestrin could be one of the numerous potential targets for therapy.

The main pathway for calcium efflux from the cell is the Na - Ca exchanger (NCX), a membrane antiporter and a determinant of both the electrical and contractile state of the heart. NCX causes efflux of one Ca(2+) for three Na+ transported into the cell. Enhanced expression of NCX has recently been recognised as one of the molecular mechanisms that contributes to reduced Ca(2+) release, impaired contractility and an increased risk of arrhythmias during the development of cardiac hypertrophy and failure. The NCX also plays a crucial role in the pathogenesis of arrhythmias and cellular injury associated with ischaemia and reperfusion. Hence, NCX blockade represents a potential therapeutic strategy for treating cardiac disease.However, its reversibility and electrogenic properties must be taken into consideration when predicting the outcome. NCX inhibition has been demonstrated to be protective against ischaemic injury and to have a positive inotropic and antiarrhythmic effect in failing heart cells [21].

Failing hearts show a delay of repolarization and prolongation of action potential due to diminished potassium currents, which is proarrhythmic. Adenovirus over- expressing the potassium channels [AdShK] can reverse the prolongation of action potential duration, thereby potentially preventing arrhythmias [22].

## Ventricular arrhythmias

Electrical alternans has been linked to the development of ventricular arrhythmias.Increasing the rapid component of the delayed rectifier current (I_Kr_) may suppress electrical alternans and may be antiarrhythmic. I_Kr_ in isolated canine ventricular myocytes was increased by infection with an adenovirus containing the gene for the pore-forming domain of I_Kr_ [human ether-a-go-go gene (HERG)]. The voltage at which peak I_Kr_ occurred was significantly less negative in HERG-infected myocytes, thereby shifting the steady-state voltage-dependent activation and inactivation curves to less negative potentials [23]. This observation lend support to the idea that increasing I_Kr_ may be a viable approach to suppressing electrical alternans thereby suppressing arrhythmias.

## Current problems with gene therapy

As alluded to earlier, gene medicine is still in its infancy. Except for a few human trials, majority of trials to date have been in experimental animals. The expected result from gene therapy is a permanent cure of arrhythmias with a single stage treatment with minimal or no adverse effects. Obviously we are far from the ideal. In the field of arrhythmias, an expectant waiting for the scenario to unfold in full is needed. The available vectors to date are far from ideal. Problems with vectors include variability in transfection capabilities,inefficient delivery at site,limited period of gene expression, and immunogenicity. The tissue expression of many genes are transient. The level and efficiency of expression of many trans genes are suboptimal. Many viral vectors are potentially immunogenic and carcinogenic. The interaction between vector and host genome can result in the vector being rendered replicant and lose the therapeutic gene. Another area of concern is that the currently available vectors have less ability to transduce vascular cells than nonvascular cells. This could hamper efforts at cardiovascular gene transfer. Traditional vectors need to be engineered to increase their affinity for the target tissue or cell and prevent transduction to other cells [24].

Succesful transfer of the therapeutic gene to all the myocytes at the target site is not fully achieved in the experimental settings. The receptors for many viral vectors are present in many tissues therby limiting the specificty of gene delivery.

Many an arrhythmia with a diffuse substrate like atrial fibrillation needs the gene to be delivered to a wide area [the whole of the atrium]. The transfer methods like direct injection into myocardium fails to deliver the gene a short distance from the injecton site. A special area of concern in arrhythmia gene therapy is the potential for the remedy itself being arrhythmogenic. Incomplete restoration may in also be arrhythmogenic. In a non linear system like biological organisms, making an isolated change in a specific aberration will result in restoration of normal function only if the defect is truly isolated and is the direct cause of the phenotypic response. The long term response of a genetic modification in the myocardium is unknown at present. Only continued research and time can answer these problems with certainty.

## The Future

Newer refinements in vector development and design are needed to have better transduction in cardiovascular tissue. Cell specific regulatory elements and promoters to selectively target the cardiac tissue is a potential area of interest [25]. Application at bed side awaits further refinement in gene delivery. Bactofection (bacterial gene delivery) as an alternative to viral vectors has been proposed [26]. Hybrid vectors, gutted vectors and new generation non viral vectors may hold the key to future research.

## Conclusion

Given a wide plethora of potential targets for gene therapeutic strategies, the possible applications are unlimited. We have still a long way to go from animal models to the level of safe and efficacious application at the bedside. This awaits more refinements in gene delivery methods and vector designs. Not to be forgotten are the increasing concerns about safety. A regulated and sustained target tissue expression of the transduced gene with a wide index of safety should be the ultimate goal of any genetic intervention.

## References

[R1] Members of the Sicilian gambit (2001). New approaches to antiarrhythmic therapy, part II. Circulation.

[R2] Lee ER, Marshall J, Siegel CS (1996). Detailed analysis of structures and formulations of cationic lipids for efficient gene transfer to the lung. Hum Gene Ther.

[R3] Armentano D, Zabner J, Sacks C (1997). Effect of the E4 region on the persistence of transgene expression from adenovirus vectors. J Virol.

[R4] Yla-Herttuala S, Martin JF (2000). Cardiovascular gene therapy. Lancet.

[R5] Smith AE (1999). Gene therapy - where are we?. Lancet.

[R6] Labhasetwar V, Bonadio J, Goldstein S (1998). A DNA controlled-release coating for gene transfer: transfection in skeletal and cardiac muscle. J Pharm Sci.

[R7] Donahue JK, Kikkawa K, Johns DC (1997). Ultrarapid, highly efficient viral gene transfer to the heart. Proc Natl Acad Sci U S A.

[R8] Rosen MR, Robinson RB, Brink P (2004). Recreating the biological pacemaker. Anat Rec A Discov Mol Cell Evol Biol.

[R9] Rajesh G, Francis J (2006). Biological Pacemakers. Indian Pacing Electrophysiol J.

[R10] Rosen MR, Brink PR, Cohen IS (2004). Genes, stem cells and biological pacemakers. Cardiovasc Res.

[R11] Qu J, Plotnikov AN, Sosunov EA (2004). Biological pacemaker implanted in canine left bundle branch provides ventricular escape rhythms that have physiologically acceptable rates. Circulation.

[R12] Glenn CM, Pogwizd SM (2003). Gene therapy to develop a genetically engineered cardiac pacemaker. J Cardiovasc Nurs.

[R13] Donahue JK, Heldman AW (2000). Focal modification of electrical conduction in the heart by viral gene transfer. Nat Med.

[R14] Bauer A, McDonald AD, Nasir K (2004). Inhibitory G protein overexpression provides physiologically relevant heart rate control in persistent atrial fibrillation. Circulation.

[R15] Fabritz L, Kirchhof P, Fortmuller L (2004). Gene dose-dependent atrial arrhythmias, heart block, and brady-cardiomyopathy in mice overexpressing A(3) adenosine receptors. Cardiovasc Res.

[R16] Murata M, Cingolani E, McDonald AD (2004). Creation of a genetic calcium channel blocker by targeted gem gene transfer in the heart. Circ Res.

[R17] Burton DY, Song C, Fishbein I (2003). The incorporation of an ion channel gene mutation associated with the long QT syndrome (Q9E-hMiRP1) in a plasmid vector for site-specific arrhythmia gene therapy: in vitro and in vivo feasibility studies. Human Gene Ther.

[R18] Mazhari R, Nuss HB, Armoundas AA (2002). Ectopic expression of KCNE3 accelerates cardiac repolarization and abbreviates the QT interval. J Clin Invest.

[R19] Ennis IL, Li RA, Murphy AM (2002). Dual gene therapy with SERCA1 and Kir2.1 abbreviates excitation without suppressing contractility. J Clin Invest.

[R20] Francis J, Sankar V, Krishnan Nair V (2005). Catecholaminergic polymorphic ventricular tachycardia. Heart Rhythm.

[R21] Hobai IA, O'Rourke B (2004). The potential of Na+/Ca2+ exchange blockers in the treatment of cardiac disease. Expert Opin Investig Drugs.

[R22] Nuss HB, Johns DC, Kaab S (1996). Reversal of potassium channel deficiency in cells from failing hearts by adenoviral gene transfer: a prototype for gene therapy for disorders of cardiac excitability and contractility. Gene Ther.

[R23] Hua F, Johns DC, Gilmour RF (2004). Suppression of electrical alternans by overexpression of HERG in canine ventricular myocytes. Am J Physiol Heart Circ Physiol.

[R24] Baker AH (2004). Designing gene delivery vectors for cardiovascular gene therapy. Progs biophys mol.

[R25] Beck C, Boren Jan, Akyurek Levent M (2004). Tissue specific targeting for cardiovascular gene transfer. Potentials vectors and future challenges. Curr Gene Therapy.

[R26] Palffy R, Gadlik R, Hodosy J (2006). Bacteria in gene therapy; Bactofection versus alternative gene therapy. Gene Therapy.

